# Integrated Analysis of microRNA and mRNA Expression Profiles: An Attempt to Disentangle the Complex Interaction Network in Attention Deficit Hyperactivity Disorder

**DOI:** 10.3390/brainsci9100288

**Published:** 2019-10-22

**Authors:** Nicoletta Nuzziello, Francesco Craig, Marta Simone, Arianna Consiglio, Flavio Licciulli, Lucia Margari, Giorgio Grillo, Sabino Liuni, Maria Liguori

**Affiliations:** 1 Institute of Biomedical Technologies, National Research Council, Bari Unit, 70126 Bari, Italy; nicoletta.nuzziello@gmail.com (N.N.); ariannaconsiglio@gmail.com (A.C.); flavio.licciulli@ba.itb.cnr.it (F.L.); sabino.liuni@ba.itb.cnr.it (S.L.); 2 Unit for Severe Disabilities in Developmental Age and Young Adults, Scientific Institute IRCCS E. Medea, 72100 Brindisi, Italy; fcraig.npi@gmail.com; 3 Child Neuropsychiatric Unit, Department of Biomedical Sciences and Human Oncology, University “Aldo Moro” of Bari, 70124 Bari, Italy; martasimone_ms@libero.it; 4 Child Neuropsychiatric Unit, University “Aldo Moro” of Bari, 70124 Bari, Italy; lucia.margari@uniba.it

**Keywords:** circulating biomarkers, microRNA, transcriptome, targetome, bioinformatics, high-throughput next-generation sequencing (HT-NGS)

## Abstract

Attention Deficit Hyperactivity Disorder (ADHD) is a childhood-onset neurodevelopmental disorder, whose etiology and pathogenesis are still largely unknown. In order to uncover novel regulatory networks and molecular pathways possibly related to ADHD, we performed an integrated miRNA and mRNA expression profiling analysis in peripheral blood samples of children with ADHD and age-matched typically developing (TD) children. The expression levels of 13 miRNAs were evaluated with microfluidic qPCR, and differentially expressed (DE) mRNAs were detected on an Illumina HiSeq 2500 genome analyzer. The miRNA targetome was identified using an integrated approach of validated and predicted interaction data extracted from seven different bioinformatic tools. Gene Ontology (GO) and pathway enrichment analyses were carried out. Results showed that six miRNAs (miR-652-3p, miR-942-5p, let-7b-5p, miR-181a-5p, miR-320a, and miR-148b-3p) and 560 genes were significantly DE in children with ADHD compared to TD subjects. After correction for multiple testing, only three miRNAs (miR-652-3p, miR-148b-3p, and miR-942-5p) remained significant. Genes known to be associated with ADHD (e.g., *B4GALT2, SLC6A9 TLE1, ANK3, TRIO, TAF1,* and *SYNE1*) were confirmed to be significantly DE in our study. Integrated miRNA and mRNA expression data identified critical key hubs involved in ADHD. Finally, the GO and pathway enrichment analyses of all DE genes showed their deep involvement in immune functions, reinforcing the hypothesis that an immune imbalance might contribute to the ADHD etiology. Despite the relatively small sample size, in this study we were able to build a complex miRNA-target interaction network in children with ADHD that might help in deciphering the disease pathogenesis. Validation in larger samples should be performed in order to possibly suggest novel therapeutic strategies for treating this complex disease.

## 1. Introduction

Attention Deficit Hyperactivity Disorder (ADHD) is a childhood-onset neurodevelopmental disorder characterized by inappropriate and impairing inattention, impulsivity, and hyperactivity [1]. The disease occurs in 2%–10% of school-age children [2], more frequently identified in young males [3]. Persistence rates of ADHD in adulthood range from 40% to 75%, with a worldwide prevalence of 3.4% [4,5]. 

ADHD is considered a complex disorder caused by environmental [6], epigenetic [7], and genetic factors [8,9]. In the general population, the risk of ADHD is estimated to be around 20% in the first-degree relatives of probands [2]. Several candidate genes [10,11,12,13] have been implicated in ADHD susceptibility, and a recent genome-wide association study (GWAS) identified significant risk loci located within or nearby genes involved in neurodevelopment processes [8]. 

To date, many critical features still need to be clarified about ADHD: the etiology and pathogenesis of the disease are still largely unknown, and its diagnosis continues to be heatedly debated, as it is based on the observation of behavioral signs and verbal reports, rather than on measurable biological parameters; moreover, personalized and efficient therapeutic approaches are missing. Given the clinical heterogeneity of ADHD and its high comorbidity with other psychopathological disorders [14], there is an urgent need to identify useful molecular signatures that help in deciphering the disease pathogenetic mechanisms, thus facilitating the diagnosis and possibly addressing new therapeutic strategies. 

Micro-ribonucleic acids (miRNAs), highly evolutionarily conserved endogenous small noncoding RNAs, have been investigated as biomarkers for the diagnosis of ADHD, as well as for monitoring its progression and response to treatment [15,16,17]. MiRNAs configure complex molecular networks where they may regulate hundreds of genes individually, and up to 80% of the human genome collectively [18,19,20]. Lines of evidence confirm the role of miRNAs in ADHD etiology, pathogenesis, and diagnosis; nevertheless, the studies are still few in number and do not overlap with each other [15,16,17,21]. The first study reported a decreased expression of five miRNAs in children with ADHD compared to controls [15]. Recently, five dysregulated serum miRNAs were identified in a more comprehensive set of 84 miRNAs, whereas the first and only global screening technology exploring the miRNA profile in ADHD was performed in the Chinese population, which identified 13 miRNAs as potential ADHD diagnostic biomarkers [16]. 

The aim of this study was to identify critical key hubs and aberrant miRNA-based regulatory networks in order to provide deeper insight into the genetic mechanisms underlying this multifactorial and complex disease, thus helping to clarify the gray areas of ADHD. To this purpose, we investigated the expression profile of several circulating miRNAs in children with ADHD employing a miRNA panel tested for the study of other neurological diseases [22]. Using a high-throughput next-generation sequencing (HT-NGS) approach, we also performed a genome-wide profiling of the mRNA fraction in order to identify differentially expressed (DE) target genes with an unbiased approach, and to search for miRNA-target interaction network(s) possibly related to ADHD.

## 2. Materials and Methods

**Figure 1:**
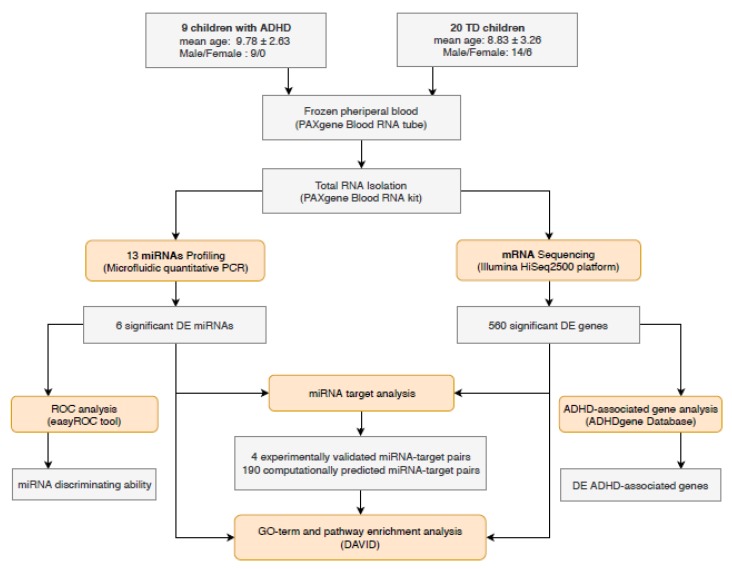
Flow chart describing the study design.

Figure 1 shows the flow chart of our study design (details below).

### 2.1. Study Population

The study recruited children diagnosed with ADHD, according to the Diagnostic and Statistical Manual of Mental Disorders (DSM-5) criteria, among those diagnosed, followed-up, and consecutively presenting at the Child Neuropsychiatric Unit, University of Bari, Italy. ADHD patients were less than 18 years old and they were not previously exposed or under concomitant psychotropic drugs. Children with a history of comorbid major psychiatric disorders, such as autism spectrum disorder, bipolar disorders, major depression, obsessive-compulsive disorders, psychotic disorders, epilepsy, or severe head injury, were excluded. 

Age-matched typically developing (TD) children were recruited from the same geographic area; they did not show clinical signs or instrumental evidence for any of the aforementioned major psychiatric disorders.

The study was approved by the Ethics Committee of Azienda Ospedaliera Policlinico, University of Bari. Written informed consent (according to the Declaration of Helsinki) was obtained from the parents or legal tutors of the children enrolled in the investigation.

### 2.2. Sample Preparation

Peripheral blood samples were collected from children with ADHD and controls and stored at –20 °C in 3 ml PAXgene Blood RNA Tubes (PreAnalytiX Qiagen/BD, Hombrechtikon, Switzerland). Total RNA was isolated using the PAXgene Blood RNA Kit (PreAnalytiX Qiagen/BD, Hilden, Germany). RNA quantity and quality were measured using Nanodrop ND-1000 spectrophotometer (Thermo Fisher Scientific, Wilmington, DE, USA) and RNA 6000 Pico chip on Bioanalyzer 2100 (Agilent Technologies, Santa Clara, CA, USA), respectively. Samples with RNA integrity number (RIN) scores higher than 7 and with A260/A280 values in the 1.8–2.2 range were processed for further analyses. 

### 2.3. miRNA Profiling: Reverse Transcription and Microfluidic qPCR

TaqMan Advanced miRNA Cards (Applied Biosystems, Thermo Fisher Scientific) were employed for miRNA quantitative analysis. Approximately 8 ng of total RNA/sample was reverse transcribed using a TaqMan Advanced miRNA cDNA synthesis kit (Applied Biosystems, Thermo Fisher Scientific). The ends of each mature miRNA were extended with 5′-end ligation of an adaptor sequence and 3′ poly-A tailing, and recognized by universal RT primers (Applied Biosystems, Thermo Fisher Scientific). The obtained cDNA was amplified using the Universal miR-Amp Primers (Applied Biosystems, Thermo Fisher Scientific), diluted, and served as a template for microfluidic qPCR analysis with TaqMan Advanced miRNA Cards (Applied Biosystems, Thermo Fisher Scientific). Briefly, 25 µL of preamplified product was mixed with 50 µL of TaqMan Fast Advanced Master Mix (Applied Biosystems, Thermo Fisher Scientific) and dispensed into each port of the TaqMan Advanced miRNA Card. The reaction was performed on ABI PrismVR 7900HT sequence detection system (Applied Biosystems, Life Technologies, Carlsbad, CA, USA) according to the parameters reported in a previous study [22]. In detail, thermal cycling parameters were 10 min at 92  °C to enzyme activation, 40 cycles of denaturation at 95 °C for 1 s, and annealing and extension at 60  °C for 20 s. 

Raw Ct-values were calculated using Expression Suite™ software v1.1 (Life Technologies, Thermo Fisher Scientific). The cycle number at which the reaction crossed an arbitrarily placed threshold (Ct) was determined for each miRNA. We used Ct = 40 as a cut-off. The relative expression levels of each miRNA, normalized to miR-191-5p and miR-93-5p (the most suitable endogenous reference miRNAs resulting from NormFinder and GeNorm tools), were calculated according to the 2^−ΔΔCt^ method, detailed in [23]. We performed the statistical analysis of miRNAs (DE) with the Expression Suite™ software, which consists of two-tailed Student’s *t*-test. The obtained *p*-value was corrected for multiple comparisons using Benjamini–Hochberg’s method. A *p*-value <0.05 was considered statistically significant.

The miRNA’s ability to discriminate the compared groups (ADHD versus TD) was determined using receiver-operating characteristic (ROC) analysis, calculated with the easyROC web-tool [24]. Statistical significance was set at *p*-value < 0.05; the Youden cut-off method was used to determine cut-off values. The associated *p*-values and area under ROC (AUC) were calculated for each miRNA. 

### 2.4. mRNA Profiling: HT-NGS

mRNA libraries were prepared using the TruSeq Stranded mRNA Sample Preparation kit (Illumina). Briefly, 1 µg of total RNA was used for poly-A mRNA selection with oligo-dT beads, followed by thermal mRNA fragmentation and reverse transcription (RT). The obtained cDNAs were 3′-end adenylated and ligated to Illumina paired-end sequencing adapters and subsequently amplified by 12 cycles of PCR. The libraries were fluorimetrically quantified and analyzed, pooled together to obtain equimolar concentrations into a multiplex sequencing pool, and sequenced to generate 2 bp × 100 bp paired-end reads (around 30 million reads/sample) using an Illumina HiSeq2500 platform. 

### 2.5. mRNA Profiling: Bioinformatic Analyses

RNA-Seq data of ADHD and TD samples were processed, according to an in-house developed bioinformatics pipeline based on the standard tools developed for NGS data elaboration: sequencing quality was assessed with FastQC [25]; mRNA reads were mapped with STAR [26]; multireads were evaluated with RSEM [27] and MultiDEA [28] tools; differential expression analysis was performed with DESeq2 [29], which used Benjamini–Hochberg for correction. The change in the expression was considered statistically significant if the adjusted *p*-value was <0.05. 

In order to identify genes closely related to ADHD, the list of DE genes, obtained through the previous pipeline, was matched to public available ADHD-associated genetic factors by using ADHDgene Database [30]. This database contains multitype genetic factors associated with ADHD (including SNPs, CNVs, VNTR, microsatellites, genes, chromosomal regions, and biological pathways) and obtained from both deep literature screening with manual curation and extended functional analyses.

### 2.6. miRNA Target Analysis

Seven publicly available tools of predicted and validated miRNA-target interactions were used: miRanda [31], DIANA-microT-CDS [32], rna22 [33], mirDB [34], and TargetScan [35] collect predicted miRNA targets; two databases, miRtarbase [36] and DIANA-TarBase [37], contain validated miRNA-target interactions.

In order to reduce the probability of false positive results, only those miRNA/mRNA bindings that were confirmed by reporter gene assays in the previously mentioned databases, or computationally predicted by at least three algorithms, were finally selected.

### 2.7. Pathway Analysis

Functional and pathway enrichment analysis of identified DE genes were performed using the Database for Annotation, Visualization and Integrated Discovery (DAVID v6.814, https://david.ncifcrf.gov/) tool and analyzed by one-tailed Fisher’s exact test followed by the Benjamini correction with a threshold *p*-value < 0.05. 

## 3. Results

This study included 9 children with ADHD with a mean age of 9.78 (SD 2.63) and 20 TD subjects with a mean age of 8.83 (SD 3.26). No statistically significant difference was detected between the two groups by age (*p* = 0.171). A gender-related discrepancy was registered, since the ADHD patients were all males compared to 14 males and 6 females in the TD group (*p* = 0.0316); to avoid any biases due to gender differences, all the comparisons were performed between the ADHD patients and both the 20 TD subjects and the 14 male TD. Since the results did not change (Table S1), we decided to show here the first set of data to increase the significance of the comparison.

The ADHD patients showed impairment of concentration, processing speed, working memory, and cognitive flexibility performances, as documented by a complete neuropsychological evaluation routinely performed at the study entry [38]. No significant interindividual variabilities were recorded among them.

### 3.1. Differentially Expressed miRNAs in ADHD

We evaluated the expression of 13 miRNAs (let-7a-5p, let-7b-5p, miR-25-3p, miR-125a-5p, miR-942-5p, miR-221-3p, miR-652-3p, miR-182-5p, miR-185-5p, miR-181a-5p, miR-320a, miR-99b-5p, and miR-148b-3p) in children with ADHD using a miRNA panel tested for the study of other neurological diseases [22]. 

**Figure 2:**
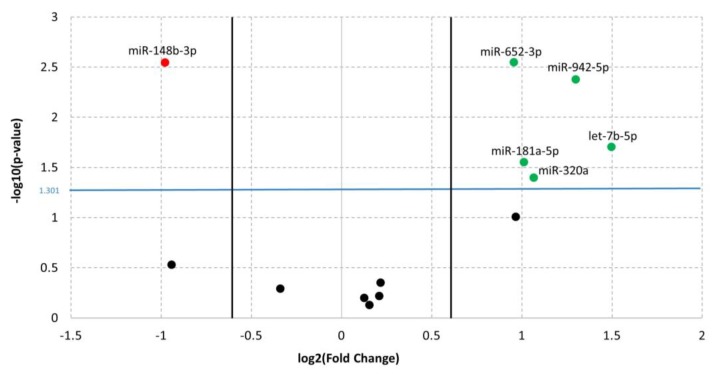
Volcano plot of qPCR data referring to the comparison of miRNAs expressions between ADHD and TDs. The Y-axis values show the negative logarithm base 10 (log10) of the p-values; the blue horizontal line on the plot represents the threshold p-value used for this analysis (0.05). The values in the X-axis indicate the log_2_ differences in estimated relative expression of the miRNAs of interest; the vertical lines represent the thresholds for the log_2_ fold change (equivalent to a fold change of 1.5). Thus, the red dot corresponds to downregulated miRNA, whereas the green dots correspond to upregulated miRNAs.

The comparison of miRNA expression levels within the study groups revealed six mature miRNAs significantly DE between ADHD and TD (Figure 2). After Benjamini–Hochberg correction for multiple testing, only miR-652-3p, miR-148b-3p, and miR-942-5p remained significant (adjusted *p*-value, adj. *p*-value < 0.05). Despite this result, given the small sample size and the functional significance of the over-threshold miRNAs, we enclosed in the analysis of the targeted mRNAs all the six miRNAs (*p*-value < 0.05) (see discussion for comments).

In detail, results from the microfluidic qPCR analysis showed statistically significant upregulation of miR-652-3p (Fold Change FC = 1.94; *p*-value = 2.84 × 10^−3^), miR-942-5p (FC = 2.46; *p*-value = 4.22 × 10^−3^), let-7b-5p (FC = 2.82; *p*-value = 1.98 × 10^−2^), miR-181a-5p (FC = 2.02; *p*-value = 2.82 × 10^−2^), and miR-320a (FC = 2.09; *p*-value = 4.02 × 10^−2^) and statistically significant downregulation of miR-148b-3p (FC = 1.97; *p*-value = 2.85 × 10^−3^) in children with ADHD compared to TDs (Table 1). 

As detailed before, in order to prevent any misleading results due to the gender ratio discrepancy between the two study groups, we ran the same analysis by comparing the miRNA expressions of the ADHD (all males) versus the 14 TD males, and we confirmed that the same six miRNAs were significantly different between the two groups (Table S1). 

**Figure 3:**
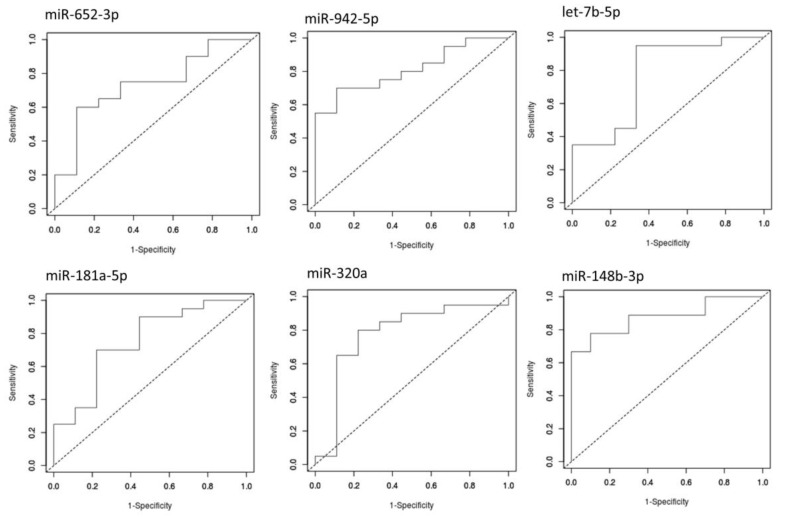
ROC curves generated by using the relative expression data of six DE miRNAs of interest. The diagram is a plot of sensitivity (true-positive rate) versus specificity (false-positive rate). AUC provides an estimate of the miRNA’s ability to discriminate between the compared groups.

To discriminate between the compared groups, we performed receiver-operating characteristic (ROC) analysis using the easyROC tool [24]. The independent miRNA analysis showed significant diagnostic values for all the DE miRNAs (*p*-value < 0.05). The six miRNAs provided values of the area under the curve (AUC) higher than 0.7, discriminating ADHD from TD (Figure 3). The best AUC values were observed for miR-320a (0.811; *p*-value =1 × 10^−4^) and miR-148b-3p (0.878; *p*-value = 5.5 × 10^−6^). 

### 3.2. Identification of DE mRNAs 

In order to obtain a list of mRNAs involved in ADHD susceptibility, differential expression analysis of RNA-Seq data between the two subgroups (ADHD vs. TD) was performed, using three criteria already tested in previous analyses [22,23]: (1) an average absolute fold change between the two study groups greater than 1.5; (2) a mean number of reads greater than 25; and (3) statistical significance (adjusted *p*-value, adj. *p*-value) lower than 0.05.

As result, 560 genes (200 upregulated and 360 downregulated) showed significant DE in children with ADHD compared to TD subjects (Table S2).

### 3.3. Target Analysis

To identify the miRNA targetome, the 560 DE mRNAs and the 6 DE miRNAs were selected for gene target analysis, using an integrated approach of validated and predicted interaction data extracted from seven different bioinformatic tools. Using databases containing experimentally validated miRNA-target interactions (miRtarbase and DIANA-Tarbase), four miRNA-target pairs, validated by reporter gene assays, were selected. Furthermore, since the prediction of the target site of existing algorithms can be still characterized by low precision and poor sensitivity, we integrated the predictions of at least three out of five miRNA-target interaction tools (miRanda, RNA22, mirDB, TargetScan, DIANA-microT-CDS) according to published guidelines [39]. 

This extensive analysis was able to uncover 190 predicted miRNA-target pairs, including 131 target genes; three miRNA-target pairs (miR-181a-5p/PHLPP2, let-7b-5p/E2F2, and let-7b-5p/IGF2BP2) were found overlapping between the experimentally validated and computationally predicted miRNA-target interactions. 

**Figure 4:**
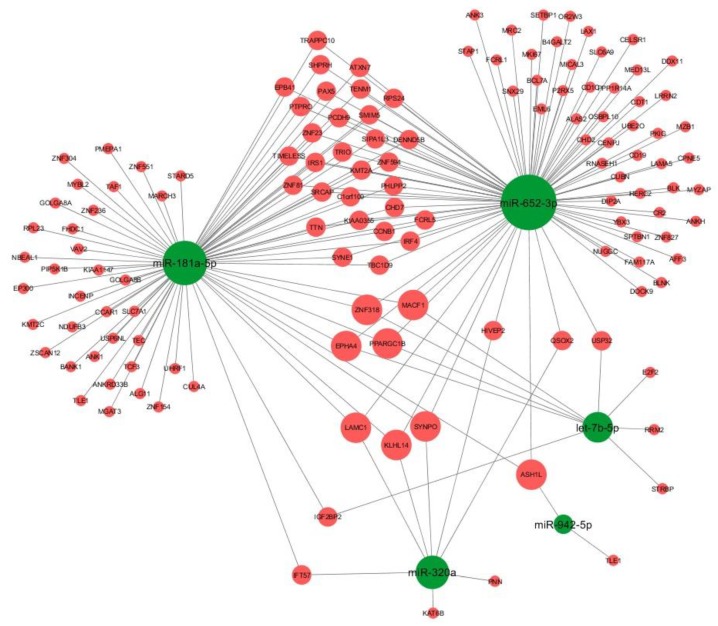
Graphical representation of miRNA-based targetome using Cytoscape v3.6.0. Only computationally predicted (three out of five algorithms) and/or validated miRNA-target interactions are shown. Green nodes represent miRNAs, red nodes represent target genes. The size of the nodes is proportional to the degree of the nodes (i.e., number of incoming and outcoming edges).

We therefore constructed the miRNA-based network (Figure 4) including the DE miRNAs and their associated DE targets using Cytoscape v3.6.0 [40]. Interestingly, eight target genes (*KLHL14, ASH1L, MACF1, ZNF318, EPHA4, LAMC1, PPARGC1B*, and *SYNPO*) were shared by three miRNAs.

The DE genes were matched to publicly available ADHD-associated genetic factors contained in the ADHDgene Database [41], and we confirmed several miRNA targets previously associated to ADHD (TLE1, ANK3, TRIO, TAF1, SYNE1). 

### 3.4. GO-Term and Pathway Enrichment Analysis

**Figure 5:**
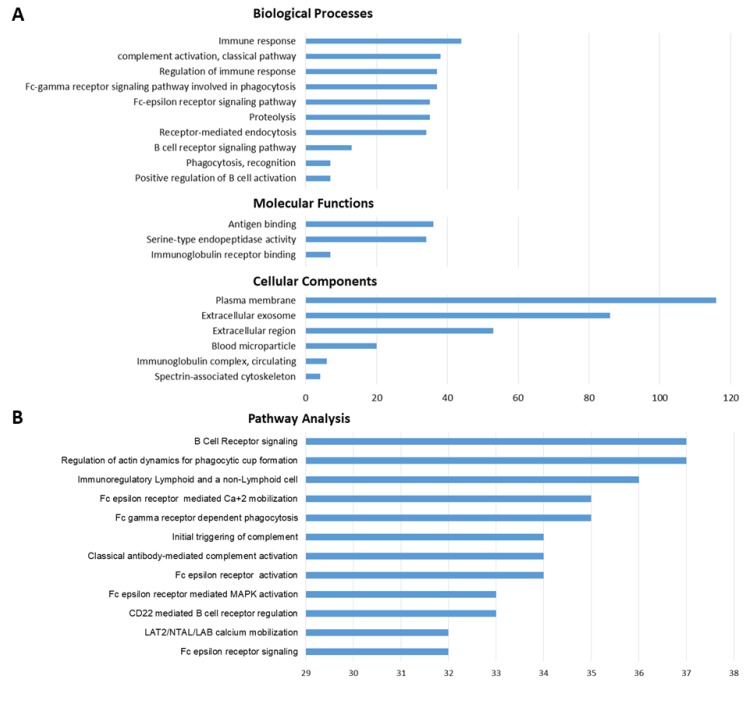
The histograms illustrate the category of enriched GO terms (**A**) and enriched pathways (**B**) for the DE genes. The horizontal axis represents the number of genes.

To explore the potentially involved physiological functions, all target genes were subjected to Gene Ontology (GO) and pathway enrichment analysis using the DAVID 6.8 Functional Annotation Tool (http://david.abcc.ncifcrf.gov/). The three GO categories (biological processes, cellular components, and molecular functions) were used to describe the gene product attributes (Figure 5A). Meanwhile, the results of pathway enrichment analysis revealed the main pathways in which the target genes were involved (Figure 5B). Almost all the GO-term results were closely related to immune system functions, such as complement activation (adj. *p*-value = 2.63 × 10^−34^), regulation of immune response (adj. *p*-value = 2.41 × 10^−23^), Fc-epsilon receptor signaling (adj. *p*-value = 3.38 × 10^−21^), antigen binding (adj. *p*-value = 2.73 × 10^−31^). The pathway analysis also revealed the enrichment of immune-related pathways, such as classical antibody-mediated complement activation (adj. *p*-value = 2.29 × 10^−35^), CD22-mediated B cell receptor regulation (adj. *p*-value = 7.46 × 10^−35^), Fc-epsilon receptor activation (adj. *p*-value = 3.83 × 10^−34^), B cell receptor signaling (adj. *p*-value = 1.10 × 10^−33^). Details of significantly implicated pathways and GO terms are reported in Table S3.

## 4. Discussion

To our knowledge, this is the first report that evaluates combined miRNA and mRNA expressions in children with ADHD. None of the three miRNAs that finally resulted DE in our analysis have been found to be implicated in the pathophysiology of ADHD, although this evidence may be explained (e.g., by some methodological bias). In fact, as in our approach, most of the studies investigated preselected miRNAs involved in other neurological disorders and/or in neurobiology mechanisms based on literature reviews or miRNA databases [15,17,21]; therefore, we may have omitted some potential miRNAs associated with ADHD, as they are involved in unknown biological mechanisms. On the other hand, Wang et al. [16] used for the first time a global screening technology (NGS) to explore miRNA profiles in ADHD of Han Chinese individuals recruited from a single site in Taiwan, but the study identified 13 different miRNAs as potential ADHD biomarkers. However, given the complex pathophysiology of ADHD, it is reasonable to hypothesize that the underlying biological mechanisms may not be identical across ethnicities. 

In our investigation, starting from a panel of miRNAs dysregulated in diseases like Pediatric Multiple Sclerosis (PedMS) [22], we identified six DE miRNAs (miR-652-3p, miR-942-5p, let-7b-5p, miR-181a-5p, miR-320a, and miR-148b-3p) in children with ADHD compared to TDs, although only three of them (miR-652-3p, miR-148b-3p, and miR-942-5p) survived from the multiple comparisons correction. The overlap of these miRNAs in ADHD and PedMS might be explained by common underlying mechanisms (e.g., their cognitive dysfunctions), since ADHD-like symptoms have been described in about 27% of PedMS cases [42]. According to the literature, miR-652-3p and miR-148b-3p were also found to be associated with autism [43]; this evidence may be justified by the clinical comorbidity between the two diseases that also seem to share some genetic background, thus complicating the differential diagnosis between them [44,45,46,47]. Furthermore, in our analysis, several DE miRNA targets were found to be associated with neuropsychiatric disorders, such as schizophrenia (TTN, SYNPO) [48,49], depressive disorder (PPARGC1B, TIMELESS) [50,51,52], and autism (TAF1, TRIO) [53,54]. Given the high overlap of DE miRNAs and mRNAs in ADHD and these disorders, it is more likely that given dysregulated miRNA-target networks may be specific to ADHD, rather than single miRNAs and/or genes. 

Using for the first time a HT-NGS approach to profile mRNA expression in ADHD, we also obtained a plethora of DE genes compared to TDs. In the resulting miRNA-targets network, the targets with the largest degree values (in terms of number of incoming and outcoming edges) were KLHL14, ASH1L, MACF1, ZNF318, EPHA4, LAMC1, and PPARGC1B, suggesting that they might be critical elements in the ADHD physiopathology. 

Several of these downregulated targets have been previously associated to ADHD, such as TLE1 [55], ANK3 [55,56], TRIO [57], TAF1 [58], SYNE1 [59]. Notably, TLE1 is essential for the maintenance of neuronal survival and its expression is reduced in neurons primed to die [60]. Since decreased subcortical volumes have been observed in ADHD patients, it is possible that a selective neuronal vulnerability is implicated in these volumetric losses [61]. Moreover, ANK3 is involved in neuronal development, playing a critical role in intellectual functioning, and its decreased expression can lead to different cognitive/psychiatric phenotypes [62]. Finally, TRIO regulates the neuronal development of the hippocampus, as well as the neuronal migration and axon guidance, by modulating RhoG that resulted in being upregulated in our analysis. Both RhoG and its regulator TRIO were found closely related to the learning functions in mice [63]. 

Recently, a genome-wide analysis identified the first significant genetic variants associated with ADHD in 12 independent loci [8]. The most strongly associated locus on chromosome 1 covered a gene-rich 250 kb region, including B4GALT2 and SLC6A9 that resulted in DE genes targeting both by miR-652-3p, one of the confirmed miRNAs in our analysis. Although the index variant was intronic to both genes, it might affect the gene expression levels in different manners. For example, variants in introns can introduce novel splice sites, activate novel promoters, introduce/eliminate enhancer activity, or modify RNA structure and, consequently, the accessibility of the target site to the RISC complex (mRNA secondary structure fold during transcription). 

In our analysis, many other identified miR-652-3p targets were related to mechanisms possibly associated with the ADHD pathophysiology. Among them, HIVEP2 has been reported in patients characterized by developmental delay and intellectual disability [64], and CHD2 was found implicated in the development of selected neural circuits (i.e., cortical and hippocampal circuits) and long-term memory [65]. KLHL14 (one of the largest degree value nodes in Figure 4) and SYNE1 have been found to be involved in GABAergic interneuron circuitry and glutamate receptor internalization at postsynaptic sites, respectively [66,67]. Glutamate and GABA are essential excitatory and inhibitory neurotransmitters in the brain, and both seem to be involved in the frontostriatal signaling as well as related to the dysfunctions of the inhibiting impulse observed in ADHD subjects [68]. Indeed, magnetic resonance spectroscopy studies showed decreased prefrontal GABA levels in children with ADHD [69]. In addition, SYNE1 deficiency is one of the most common genetic causes of cerebellar ataxia, which may be of interest given the reported evidence of the cerebellum involvement in the pathophysiology of ADHD [70]. Cerebellar symptoms have been associated with difficulties in spatial working memory in children and adolescents with ADHD [71] and variability in their reaction time, which represents one of the candidate endophenotypes in this disorder [72]. 

Finally, the GO and pathway enrichment analysis of all the DE genes in our study reported their prevalent involvement in immune functions, such as complement activation, regulation of immune response, B cell receptor signaling, innate immunity, complement cascade, and adaptive immunity. Although from a limited number of cases, these results support the hypothesis that an immune imbalance may contribute to ADHD etiology, possibly requiring a predisposing genetic background [73], or it may be at least partially causative of the clinical symptoms [74,75,76]. Interestingly, several significant enriched pathways pointed to the Fc-epsilon receptor, a key player in adaptive immunity and immediate allergic reactions through the binding of immunoglobulin antibodies that recognize an immune insult and elicit an inflammatory response with the production of cytokines. If confirmed in independent evaluations, this finding may generate novel hypotheses to explore in the pathogenesis of ADHD, with the potential to improve the search for more efficient targeted treatments.

## 5. Conclusions

In conclusion, in this study we were able to draw a genetic profile of juvenile ADHD that may help in deciphering the disease’s pathogenesis. In spite of several already mentioned limitations, we believe in fact that our findings might provide preliminary but solid suggestions about some molecular networks that may be evoked during the occurrence of the disease. A larger cohort study and additional experimental approaches (e.g., protein level of mRNA targets) need to be performed to confirm these preliminary results.

## Figures and Tables

**Table 1:** List of significant dysregulated miRNAs. For each miRNA, the log_2_FC, *p*-value, and the corresponding adjusted *p*-value from qPCR analysis have been detailed. The ROC section shows the results of AUC and associated *p*-value. The total number of miRNA targets (experimentally validated by reporter gene assays or computationally predicted by at least three algorithms) and the list of previously ADHD-associated target genes have been indicated.

miRNA	Regulation	qPCR			ROC		Target	ADHD-Associated Target Genes			
		log_2_FC	*p*-Value	adj. *p*-Value	AUC	*p*-Value					
miR-652-3p	up	0.95594	2.84 × 10^-3^	1.83 × 10^-2^	0.733	2.33 × 10^-2^	89	*B4GALT2, ANK3, SLC6A9*			
miR-148b-3p	down	−0.97755	2.85 × 10^-3^	1.83 × 10^-2^	0.878	5.46 × 10^-6^	8				
miR-942-5p	up	1.29942	4.22 × 10^-3^	1.83 × 10^-2^	0.811	1 × 10^-4^	2	*TLE1*			
let-7b-5p	up	1.49735	1.98 × 10^-2^	6.45 × 10^-2^	0.772	1.16 × 10^-2^	9				
miR-181a-5p	up	1.01275	2.82 × 10^-2^	7.33 × 10^-2^	0.75	1.82 × 10^-2^	75	*TAF1, TRIO, SYNE1*			
miR-320a	up	1.06673	4.02 × 10^-2^	8.72 × 10^-2^	0.778	1.16 × 10^-2^	8				

## Supplementary Materials

The following are available online at https://www.mdpi.com/2076-3425/9/10/288/s1, Figure S1: title, Table S1: title, Video S1: title. Table S1: List of significant dysregulated miRNAs in ADHD (all males) compared to 14 TD males, Table S2: Datasets of DE mRNAs and miRNAs, Table S3: Lists of significant GO categories and pathways associated with ADHD.
